# Genomic Breakpoints’ Characterization of a Large *CHEK2* Duplication in an Italian Family with Hereditary Breast Cancer

**DOI:** 10.3390/diagnostics12071520

**Published:** 2022-06-22

**Authors:** Aldo Germani, Daniele Guadagnolo, Valentina Salvati, Caterina Micolonghi, Rita Mancini, Gioia Mastromoro, Soha Sadeghi, Simona Petrucci, Antonio Pizzuti, Maria Piane

**Affiliations:** 1 Department of Clinical and Molecular Medicine, Sapienza University of Rome, 00185 Rome, RM, Italy; aldo.germani@uniroma1.it (A.G.); rita.mancini@uniroma1.it (R.M.); simona.petrucci@uniroma1.it (S.P.); maria.piane@uniroma1.it (M.P.); 2 Department of Experimental Medicine, Sapienza University of Rome, 00185 Rome, RM, Italy; caterina.micolonghi@uniroma1.it (C.M.); gioia.mastromoro@uniroma1.it (G.M.); soha.sadeghi@uniroma1.it (S.S.); antonio.pizzuti@uniroma1.it (A.P.); 3 Scientific Direction, IRCCS Regina Elena National Cancer Institute, 00128 Rome, RM, Italy; salvati.sv@gmail.com; 4 S. Andrea University Hospital, 00189 Rome, RM, Italy; 5 Medical Genetics Unit, IRCCS Mendel Casa Sollievo della Sofferenza, 71013 San Giovanni Rotondo, FG, Italy

**Keywords:** *CHEK2*, hereditary breast cancer, intragenic duplication, large rearrangement

## Abstract

*CHEK2* (checkpoint kinase 2; MIM# 604373) is a tumor suppressor gene that encodes a serine threonine kinase involved in pathways such as DNA repair, cell cycle arrest, mitosis, and apoptosis. Pathogenic variants in *CHEK2* contribute to a moderately increased risk of breast and other cancers. Several variant classes have been reported, either point mutations or large intragenic rearrangements. However, a significant portion of reported variants has an uncertain clinical significance. We report an intragenic *CHEK2* duplication, ranging from intron 5 to intron 13, identified in an Italian family with hereditary breast cancer. Using long range PCR, with duplication-specific primers, we were able to ascertain the genomic breakpoint. We also performed a real-time PCR to assess a possible loss-of-function effect. The genomic characterization of large intragenic rearrangements in cancer susceptibility genes is important for the clinical management of the carriers and for a better classification of rare variants. The molecular definition of breakpoints allows for the prediction of the impact of the variant on transcripts and proteins, aiding in its characterization and clinical classification.

## 1. Introduction

Hereditary cancer susceptibility conditions are considered among the major fields of work and research for clinical and molecular geneticists. The role of heterozygous point mutations and intragenic rearrangements of high penetrance genes *BRCA1* and *BRCA2* in cancer susceptibility (especially, but not only, breast and ovarian cancer) is well established, and international guidelines addressing molecular testing indications and clinical management are available [1]. Variants in other genes with high and moderate penetrance whose products are involved in the same DNA damage sensing and repair pathway can result in similar cancer susceptibility conditions [2]. *CHEK2* (checkpoint kinase 2; MIM# 604373) is a tumor suppressor gene encoding a serine–threonine kinase involved in DNA repair, cell cycle regulation, senescence and apoptosis [3]. Pathogenic variants of the *CHEK2* gene are associated with a moderately increased risk of malignancies, including, but not exclusively, breast cancer [4]. The cancer susceptibility inheritance shows an autosomal semidominant pattern, as homozygous carriers tend to present with tumors with an earlier onset and higher frequency [3]. Single nucleotide variants, small insertions or deletions and large intragenic rearrangements have been described as being damaging mutations in *CHEK2* in familial cancer cases [5,6]. A founder effect has been proposed for variants, such as the c.1100del occurring at high rates in several populations of different origins including Eastern Europeans, frequently in Poland [7], and the exon 9–10 deletion, the most common truncating *CHEK2* variant in Polish [8], Czech, and Slovakian populations [9,10]. Unfortunately, a large amount of identified variants is reported and classified as Variants of Uncertain Significance (VUS) according to the American College of Medical Genetics and Genomics (ACMG) criteria [3,11,12]. Critical uncertainties concern the identification of large intragenic rearrangements, and especially duplications [13]. It is vital to assess that the duplicated regions lie within the gene (thus possibly disrupting its function) and not in other regions of the genome [13]. Deleterious duplications are usually in tandem within the gene [13]. Assessing the position of the duplicated region can be crucial for variant classification [13]. The genomic characterization of these variants is often not performed, and data concerning effects on transcripts and protein are lacking. These studies would be extremely beneficial for variant interpretation, as *CHEK2* is a dosage-sensitive gene, and haploinsufficiency and loss-of-function mutations are known deleterious mechanisms [14]. As a further concern, it is unclear as to whether large rearrangements resulting in a haploinsufficient allele lead to higher or different cancer susceptibility risks when compared to variants with a less marked loss-of-function, as it has been suggested for other variant classes [15]. The increasing accessibility for molecular testing for moderate-risk genes like *CHEK2* poses significant concerns for all professionals involved. There are currently no guidelines addressing indications to test these genes in individuals or families, and the clinical management of a molecular diagnosis is often difficult due to the lack of genotype–phenotype correlations. Approaches detecting single- or multi-exon deletions or duplications are not always readily available, and the management of such variants is often even more complex due to the lack of molecular and clinical characterization.

We report the identification of an intragenic *CHEK2* duplication in a woman ascertained for her personal and familial history of breast and ovarian cancer. We describe the route undertaken to provide a genomic characterization of the rearrangement to assess its damaging effect on gene products. along with the studies performed on the transcript.

## 2. Case Presentation

**Figure 1:**
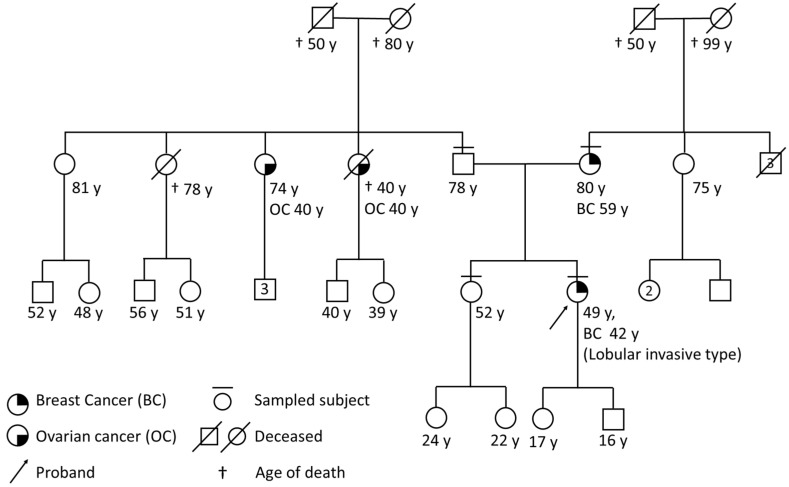
**Four-generation pedigree of the family.** The proband, III:11, was diagnosed with invasive lobular breast carcinoma at age 42. Her mother, II:6, was diagnosed with invasive ductal breast carcinoma at age 59. The proband’s paternal aunts II:3 and II:4 had ovarian cancer at age 40.

The proband (III:11 Figure 1) was an Italian 48-year-old woman undergoing genetic counseling to evaluate her eligibility for *BRCA1/BRCA2* molecular testing. She was referred for her personal and familial history of breast cancer, and for the occurrence of ovarian cancer cases in the paternal kindred. She received a diagnosis of invasive lobular carcinoma at age 42 and she reported that her 79-year-old mother (II:6, Figure 1) presented with invasive ductal carcinoma at age 59. She also reported that two paternal aunts (II:3 and II:4) underwent hysterectomy and bilateral salpingo-oophorectomy for ovarian cancer at age 40. She was eligible for *BRCA1/BRCA2* molecular analysis.

## 3. Results

### 3.1. NGS and MLPA Results for BRCA1/2 Genes

All the subjects who participated in the study gave their written informed consent during genetic counseling.

Molecular analysis of the *BRCA1/2* genes includes, as part of the routine genetic testing, screening for single nucleotide variants (SNVs), small insertions or deletions (indels) by Next Generation Sequencing (NGS) as well as Copy Number Variations (CNVs) by MLPA (Multiplex Ligation Probe Amplification) was performed.

The mutational analysis of the *BRCA1* and *BRCA2* genes did not detect pathogenic variants either with the NGS analysis or with the MLPA.

**Figure 2:**
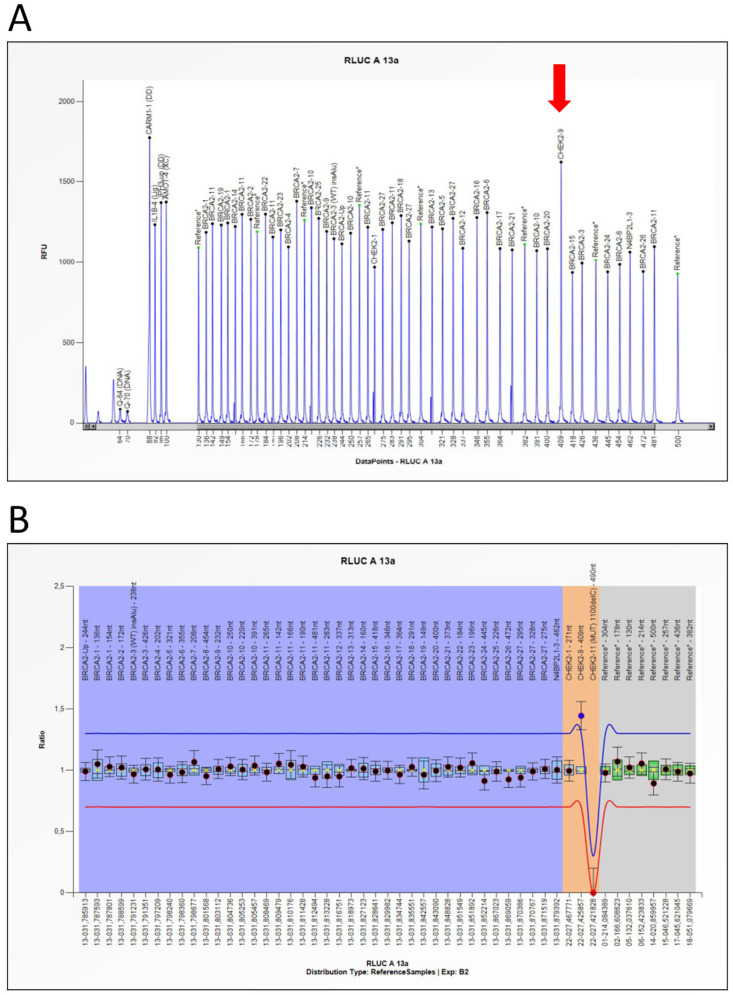
***BRCA2/CHEK2* MLPA analysis.** (**A**) *BRCA2/CHEK2* P045-D1 probemix MLPA electropherogram profiles. Red arrow shows the duplication exon 9 of the *CHEK2* gene. (**B**) MLPA Probemix P045-D1 *BRCA2/CHEK2* contains 51 MLPA probes with amplification products between 130 and 500 nucleotides (nt) and three probes for the CHEK2 gene. The blue rhombus represents the 95% confidence interval over the reference samples for each probe. The collected data were analyzed using Coffalyser.NET Software (MRC Holland).

However, the kits used for CNV detection *BRCA1* P002-D1 and *BRCA2/CHEK2* P045-D1 (MRC Holland, Amsterdam the Netherlands), featuring also probes for some regions where pathogenetic variants of the *CHEK2* gene occur, identified an apparent duplication of exon 9 of the *CHEK2* gene in the proband (Figure 2). The probes exploring exon 9 showed a relative peak ratio (RPR) value of about 1.5 when compared to the normal range value of reference probes (normal range: 0.7–1.3). This result was consistent with a duplication encompassing exon 9 of *CHEK2*, without further information on its extension.

### 3.2. Large Rearrangement Characterization on CHEK2 Gene

**Figure 3:**
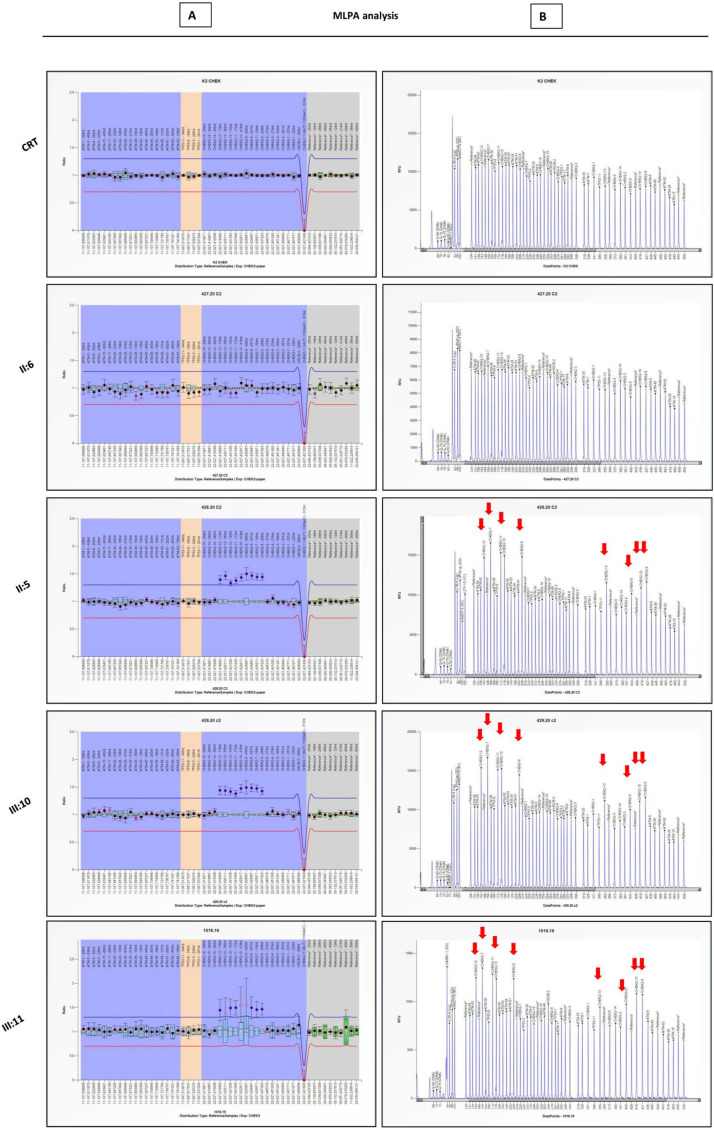
***CHEK2* MLPA with *CHEK2* P190-D1 kit on proband and first-degree relatives.** (**A**) In the proband (III:11), father (II:5) and sister (III:10), the probes for exons 6–13 show a RPR value of 1.5, greater than normal range (0.7–1.3 red and blue line), consistent with a multi-exon duplication; in the mother (II:6) all probes values fall within the normal range (0.7–1.3), comparable to the control (CRT). The blue rhombi represent 95% confidence intervals over the reference samples for each probe. (**B**) Electropherograms showing the MLPA probes for *CHEK2* exons in a reference sample and family members. Duplicated exons are indicated by red arrows. The analysis was carried out with the Coffalyser.NET Software (MRC Holland) on reference transcript for *CHEK2*: NM_007194.4.

To confirm and better determine the extent of the duplication, a more comprehensive MLPA assay of the gene was performed, using a specific kit exploring all exons (1–15) of *CHEK2* (P190-D1). This allowed the identification of a large rearrangement in the *CHEK2* gene encompassing exons 6–13 (Figure 3) on the proband (III:11). The molecular analysis of first-degree relatives confirmed the inheritance of the variant from the unaffected father (II:5) and showed its presence in the asymptomatic sister (III:10). The duplication was absent in the mother (II:6), who was affected with post-menopausal breast cancer (Figure 3). Given the high prevalence of sporadic breast cancer in post-menopausal women, it can be considered to be a possible phenocopy. Other individuals were not available for molecular analysis, and the possible segregation of the variant in the living aunt with ovarian cancer could not be confirmed.

### 3.3. Characterization and Identification of Breakpoint Duplication

**Figure 4:**
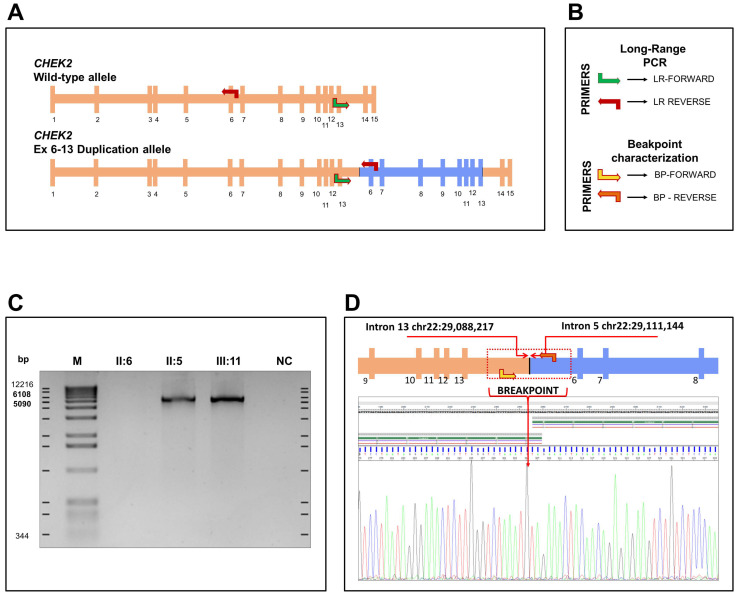
***CHEK2* exon 6–13 duplication testing and breakpoint identification.** (**A**) Schematic representation of a wild type *CHEK2* allele structure and mutant allele, with exon 6–13 tandem duplication. Red and green arrows represent reverse and forward primers, respectively, for long-range PCR. (**B**) Schematic representation of the primers for long-range PCR and of the primers for the characterization of the breakpoints. (**C**) Long-range PCR. A specific product of approximately 6 kb was detected in the proband (III:11) and in the father (II:5), and was absent in the mother (II:6). (**D**) Electropherogram showing the breakpoint sequence and schematic representation of the duplicated allele and the forward (light orange) and reverse (dark orange) primers for breakpoint sequence. The AGAT tetranucleotide aligns either to the end of intron 13 breakpoint or to the first nucleotides of the intron 5 segment. (Sequencing Analysis v5.4 software). M, molecular weight marker 1 kb (Invitrogene, Waltham, MA, USA); LR-Forward and LR-Reverse: primers used for long Range-PCR; BP-Forward and BP_Reverse: primers used for breakpoint determination.

The duplication including exons 6 to 13 identified by MLPA analysis on *CHEK2* was confirmed by long range PCR. For this purpose, the forward primer (LR-Forward) on intron 12 and the reverse primer (LR-Reverse) on intron 6 were designed as reported in Tedaldi et al., 2014 [6]. In Figure 4, the primer pairs that allowed us to characterize the duplication and the breakpoint are represented. An amplification is expected only if the duplication is in tandem. Agarose gel electrophoresis of PCR products showed a specific band of approximately 6 kb in samples II: 5 and III: 11, carrying the large rearrangement identified by MLPA analysis (Figure 2 and Figure 4), which is absent in the normal intrafamilial control II: 6 (Figure 4C). This result confirmed that the *CHEK2* duplication including exons 6 to 13 was in tandem.

**Figure 5:**
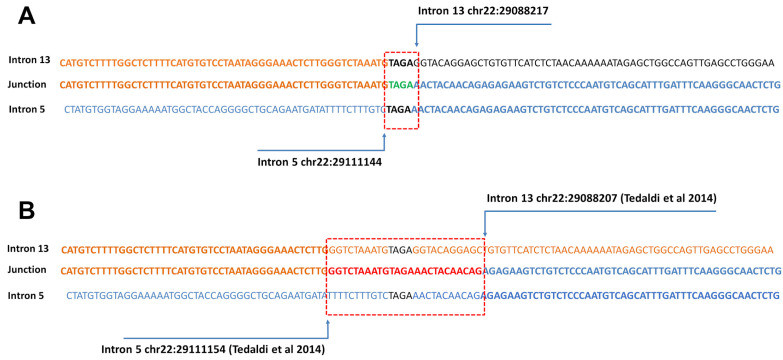
**Sequence of exon 6–13 *CHEK2* duplication.** (**A**) Aligned intron 5 and intron 13 sequences at a duplication junction identified in the proband III:11. The region of homology across the duplication is boxed. (**B**) The panel shows the different breakpoints of the rearrangement reported in the previous paper by Tedaldi et al. [6].

To determine the breakpoint, assuming that the duplication had the same breakpoint reported in Tedaldi et al. [6], a pair of primers at ±300 bp from the presumed junction was drawn. Sequence analysis showed that the duplicated region extended from intron 5, position 29,111,144, to intron 13, position 29,088,217 (NCBI RefSeq: NC_000022.10), with a tetranucleotide AGAT element overlapping the two introns (Figure 4D). Despite being very close, the duplication breakpoints of the *CHEK2* gene do not overlap with those described by Tedaldi et al. [6] (Figure 5). A bioinformatic analysis on the UCSC genome browser excluded repeated sequences within introns 5 and 13 (https://genome.ucsc.edu/, last accessed on 20 April 2022).

### 3.4. RNA Analysis

**Figure 6:**
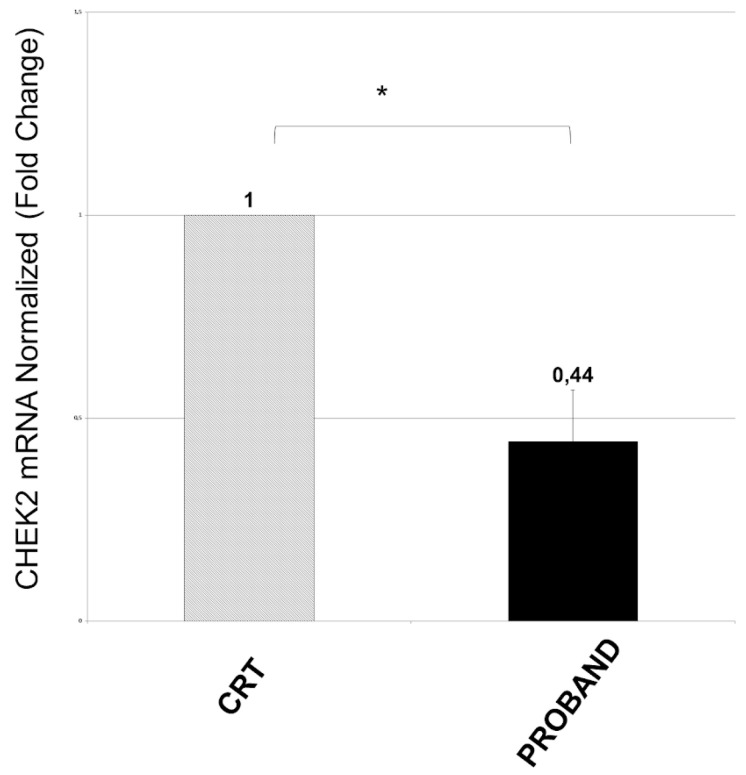
**q-PCR analysis.** Quantitative analysis of *CHEK2* cDNA from the proband showed a value of 0.44 fold change to that of a control (CRT). β-actin was used for normalization. Statistical analysis was performed using the Student’s T-tail test; * *p* < 0.05.

*CHEK2* is a dosage-sensitive gene, and quantitative RNA analyses can be performed to assess variant deleteriousness [14] The impact of exon 6–13 duplication of the *CHEK2* gene at the RNA level was evaluated by real time PCR (q-PCR) on the RNA of the proband and control by means of a pair of primers built over the exons 6/7 (Forward) and exon 11 (Reverse). The cDNA with oligodT used subsequently for q-PCR was obtained from the total RNA. This allowed us to quantify the *CHEK2* mRNA on the proband carrying the duplication and on normal controls in triplicate. The result of the analysis indicated a 44% lower amount of mRNA in the proband than in the control, suggesting an absence of expression of the allele carrying the duplication (Figure 6).

## 4. Materials and Methods

### 4.1. Mutational Analysis of BRCA1 and BRCA2 Genes

#### 4.1.1. NGS Analysis

Genomic DNA, extracted from peripheral blood of the proband and available family members by a commercially available kit (Invitrogen), Pure link Genomic DNA by Thermo Fisher Scientific, has been quantified using the Qubit ds DNA HS Assay Kit on Qubit 3.0 Fluorimeter (Invitrogen).

The Oncomine BRCA Assay was used to detect SNVs and indels on *BRCA1/2* genes. According to the manufacturer’s protocol, libraries were prepared by emulsion PCR and sequenced on the Ion Personal Genome Machine (Ion PGM^TM^) (Thermo Fisher Scientific, Carlsbad, CA, USA), using the Ion 318^TM^ Chip v2 BC. Sequencing data analysis was performed using Torrent Suite version 5.0.5 and Ion Reporter version 5.16 (Thermo Fisher Scientific).

#### 4.1.2. MLPA Analysis

Large rearrangements analysis of the *BRCA1*/*2* genes were performed by MLPA using the P002-D1 *BRCA1*, P045-D1 *BRCA2/CHEK2* (MRC-Holland, Amsterdam, Netherlands). The P045-D1 kit includes 40 probes for *BRCA2* and three probes for *CHEK2:* one for exon 1, one for exon 9, and one probe specific for the *CHEK2* c.1100delC variant, which will only generate a signal when the mutation is present. DNA samples were diluted to the final concentration of 50 ng/μL, and four normal controls were included in each MLPA assay according to the manufacturer’s instructions. Fragment analysis was performed with SeqStudio (Thermo Fisher) with size-standard 500 Liz with GeneScan^®^ Analysis Software (Thermo Fisher Scientific). The variations in peak areas were analyzed using Coffalyser.Net Software (MRC-Holland, Amsterdam, The Netherlands).

### 4.2. Duplication Analysis of CHEK2 Gene

To evaluate the extent of duplication on *CHEK2* gene, identified as an incidental finding with the P045-D1 *BRCA2/CHEK2* kit, we used a specific kit for *CHEK2* P190-D1 (MRC-Holland, Amsterdam, The Netherlands) containing 53 probes for the detection of large rearrangements in the *ATM*, *TP53* and *CHEK2* genes. In particular, for *CHEK2* gene analysis the kit includes 19 probes, spanning from exon 1 to exon 15, and one probe for the detection of the c.1100del small deletion.

### 4.3. Long-Range PCR and Breakpoint Analysis

To establish the tandem orientation of the duplicated fragment, we performed a long range (LR) PCR on the genomic DNA of the proband using a pair of primers reported by Tedaldi et al. [6] (In Figure 4A are reported as LR-Forward and LR-Reverse), with Platinum Taq DNA Polymerase High Fidelity (Thermo Fisher Scientific, Carlsbad, CA, USA), following these thermal cycling conditions: an initial denaturation at 95 °C for 2 min, 30 cycles at 98 °C for 30 s, 72 °C for 5 min and a final elongation at 72 °C for 5 min.

Assuming that the breaking points were the same as those reported by Tedaldi et al., (2014), to identify the breakpoint, a further pair of primers, both closer to the hypothetical junction, was designed: BP-F: 5′ tctgttccaatcctctcaac 3′ and BP-R 5′ tgtatggtatgaggtaagggtc 3′ (Figure 4A). The amplified template was analyzed by Sanger sequencing, performed with the BigDye Terminator 3.1 cycle sequencing kit on a SeqStudio sequencer (Thermo Fisher Scientific, Carlsbad, CA, USA), according to the manufacturer’s instructions.

### 4.4. RNA Extraction and qPCR

Total RNA was extracted from the RNeasy Mini kit (Qiagen, Hilden, Germany) according to the manufacturer’s instructions. RNA quantity and quality were determined by a NanoDrop, and 100–200 ng RNA was converted into cDNA by the iScript cDNA Synthesis Kit (BioRad, Hercules, CA, USA) with Oligo(dT) primers protocol. The cDNA was used for q-PCR experiments carried out on a Stratagene Mx3000P (Agilent Technologies, Santa Clara, CA, USA) and performed by SYBR-GREEN Gene Expression Assay (Meridian Bioscience, Cincinnati, OH, USA). We designed *CHEK2* forward primers that span the exon–exon boundaries on exon 6 and 7 and reverse primers for exon 11. The relative amount of all mRNAs was calculated using the comparative method (2−∆∆Ct) after normalization to a housekeeping gene (β-actin).

Primers for real-time PCR were as follows: *CHEK2* Forward Ex6/7 5′-AGAGGCAGACCCAGCTCTC-3′, Reverse Ex11 5′- GGTTCCACATAAGGTTCTCATGA-3′; three independent experimental replicates were performed.

## 5. Discussion

*CHEK2* is a 54-kb-gene, mapping on 22q12.1 on the reverse strand (chr22:29083730-29137821, hg19). Its canonical transcript, NM_007194, has 15 exons. It encodes for a 543-amino acid, 65 kDa serine/threonine kinase implicated in the double-strand DNA damage sensing pathway [3]. The protein is referred to as CHEK2 or CHK2. It regulates checkpoint-mediated cell cycle arrest, DNA repair and DNA-damage-induced apoptosis or senescence [3]. This pathway is shared with many proteins encoded by genes whose germline mutations are often implicated in hereditary cancer susceptibility, such as *ATM*, *BRCA1* and *BRCA2* [1]. When DNA double-strand breaks are sensed by the DNA repair machinery, the ATM protein phosphorylates CHEK2 at Threonine 68, inducing its dimerization and activation [4]. Downstream targets include key effectors mediating DNA repair and cell cycle control, such as TP53, BRCA1 and CDC25 [3].

Heterozygous *CHEK2* pathogenic variants are implicated in hereditary susceptibility to different cancer types (Prostate, MIM#176807; Colorectal, MIM#114500; Breast MIM#114480) with an autosomal dominant inheritance pattern, reduced penetrance (some individuals harboring the variant do not present related phenotypes) and variable expressivity (different individuals with the same variant may present different phenotypes). Biallelic pathogenic variants might determine a higher risk of cancer with an earlier onset [3], but this assumption is questioned [5]. The lifetime risk for breast cancer is 23–48% for female heterozygous carriers and 0.1/1% for males [16]. The risk for prostate and colorectal cancer appears to be increased when compared to the general population, but specific risk figures are lacking. The association with ovarian, thyroid and renal cancer has been demonstrated in some studies, but debated in other ones [16,17]. The identification of the c.1110del in cases of Li-Fraumeni syndrome with no *TP53* pathogenic variant led researchers to suspect *CHEK2* as the possible cause of Li-Fraumeni syndrome 2 (MIM#609265) [18]. Subsequent studies questioned the evidence [19,20], but recent reports still imply that a possible contribution of *CHEK2* variants to Li-Fraumeni-like phenotypes cannot be excluded [21]. Genotype–phenotype correlations seem to suggest that damaging variants resulting in a marked loss-of-function are associated with a higher risk of neoplasms, usually with early onset when compared to variants with a milder effect [5].

Variants belonging to different molecular classes have been described in *CHEK2*: single-nucleotide substitutions, small insertions and/or deletions, gross intragenic rearrangements [5,6]. Truncating variants and a small number of missense variants, such as p.Arg117Gly, appear to have a higher penetrance when compared to most missense variants [5]. To the best of our knowledge, the penetrance of large intragenic duplications has not been evaluated or compared to that of other variants. Unfortunately, due to the reduced penetrance and variable expressivity of the cancer susceptibility condition, the absence of large families available for molecular testing and the lack of functional studies, most variants are classified as VUS [2]. Recently, adjustments of the ACMG variant classification were proposed for *CHEK2* [12]. As of 9 April 2022, the ClinVar database (https://www.ncbi.nlm.nih.gov/clinvar/) [22] reported 2922 variants, of which 1520 (52%) were VUS. Only nine intragenic duplications > 1 kb were reported, none of them involving the same exons as the one we report. Of the duplications, six were classified as VUS (ClinVar Variant ID 240748, 653508, 583775, 583764, 584001, 216644), two as Likely Pathogenic (Clinvar Variant ID 583911, 530264), and one as Pathogenic (ClinVar Variant ID 986728). For the latter variant (NG_008150.2:g.14927_51132dup), the ClinVar record reports supporting information for a loss-of-function effect.

Loss-of-function is the main mechanism underlying variant pathogenicity for *CHEK2*, which is considered to be a dosage-sensitive gene [23,24]. Nonsense-mediated decay might prevent aberrant RNAs from maturation in large intragenic rearrangements [25]. The RNA study we performed to assess the deleterious effect of the variant suggests that no mature mRNA is produced from the variant-bearing allele, which is functionally null (Figure 6). This result can be important for the interpretation of the pathogenicity of intragenic duplications, as some of them might produce transcripts which avoid removals and undergo post-translational modifications, resulting in a residual, possibly aberrant protein production. The demonstration of the absence of a functional allele is a cost- and time-efficient method to guide pathogenicity interpretation in those cases with intragenic *CHEK2* duplications, and a broader application might help reduce the unfortunately high rate of VUS.

The other studies we performed explored variant pathogenicity indirectly, by means of genomic characterization. MLPA is a quantitative analysis informing the copy number of target genomic regions, such as individual exons, but does not identify whether a duplicated region lies within the gene or in another genomic region [13]. The demonstration that a duplication is in tandem is crucial to assess its pathogenicity, and this problem has been addressed several times in cancer susceptibility genes [13,26]. The demonstration of the tandem orientation of the rearrangement and the quantitative RT-PCR study results strongly support the pathogenicity of the exon 6–13 intragenic duplication we identified.

A possibly pathogenic intragenic *CHEK2* tandem duplication encompassing exon 6–13 had already been identified by Tedaldi et al. in an unrelated Italian family [6]. In the three-generation pedigree provided, there were three cases of breast cancer, a case of prostate cancer, a case of ovarian cancer and a case of leukemia. The variant was confirmed in two of the individuals who had developed breast cancer. Other individuals with cancer history were not available for analysis. The authors demonstrated breakpoints in intron 5 (chr22:29111154, hg19) and Intron 13 (chr22:29088207) [6], however, despite the exons involved, the break points were different from the those found in the family we described, ruling out a possible founder effect in Italy.

Of the individuals known to bear the variants in the family we describe, only the proband (III:11, Figure 1) displayed cancer history. This is consistent with the reduced penetrance described in carriers of pathogenic *CHEK2* variants [3].

Moreover, it cannot be excluded that the proband, unlike the sister, may share with the affected mother other genetic and non-genetic susceptibility factors for breast cancer, that may act as phenotypic modifiers for breast cancer development, together with the paternal duplication.

Two sisters of the father of the proband (II:3, II:4) developed ovarian cancer, but unfortunately they were not available for analysis. The association of *CHEK2* variants and ovarian cancer susceptibility has been reported but it is still debated to date [3,16]. The c.470T>C, p.(Ile157Thr) variant, commonly referred to as the I157T allele, has been associated with different ovarian neoplasms, such as cystadenomas, borderline cancers, and low-grade invasive cancers [27]. However, other studies do not confirm the association between ovarian cancer and the c.1100del orI157T CHEK2 variants [28]. At present, the evidence is not sufficient to establish or reject the association between *CHEK2* germline variants and ovarian cancer. Interestingly, a case of ovarian cancer in a 40-year-old woman was also reported in a family harboring the *CHEK2* exon 6–13 duplication, described by Tedaldi et al., but even in that case segregation analysis could not be performed [6].

Given the molecular diagnosis and the familial ovarian cancer history, the proband III:11, her unaffected sister III:10 and father II:5, each carrying the variant, were included in a tailored prevention program. The surveillance included breast, ovarian and colorectal cancer screening for the proband and her sister, and colorectal and prostate cancer for their father.

It is to be noted that the initial identification of an intragenic *CHEK2* rearrangement was an incidental finding in a *BRCA1* and *BRCA2* analysis, due to the inclusion of exon 9 of *CHEK2* in the MLPA kit for the *BRCA1* and *BRCA2* genes. This observation highlights open questions in clinical genetics and molecular diagnostics. First, the possibility of such findings is to be discussed with the patient at pre-test counseling, but clinicians and counselors might not have access to the test specifications required to understand all the possible incidental findings. Second, targeted molecular testing heavily depends on the manufacturer’s design. The choice of the proper test depends on the knowledge of the molecular mechanisms of the specific inherited conditions, but also on the laboratory’s capability to further characterize possible incomplete findings. A last controversy concerns test indication appropriateness, as the initial clinical indication was to *BRCA1* and *BRCA2* testing only. *CHEK2* and other genes associated with a moderate cancer risk are seldom included in first-line cancer susceptibility molecular testing, and there are no specific guidelines on the accession criteria for second-tier testing. In addition, only some laboratories offer a quantitative analysis for the identification of large rearrangements, either by NGS or by MLPA. Concerning future perspectives, whole genome sequencing (WGS) appears as a promising tool. WGS allows the genome-wide identification of both SNVs and CNVs, also providing information on the orientation of the identified rearrangements [29]. In WGS, CNVs are usually inferred by algorithms analyzing read depth [29]. WGS has a very high sensitivity towards the detection of CNVs larger than 1 kb, outperforming even current gold-standard array-based technologies [29]. There is, however, a significant rate of false-positives, so results should be validated with high-specificity approaches. As a further limit, the reliability of depth-based algorithms is reduced for CNVs < 1 kb [29]. The inclusion of a wider array of genes in first-line testing, and the simultaneous detection of SNVs and CNVs are desirable to improve molecular diagnostics and to provide data for cancer susceptibility molecular genetics research. However, this should drive a parallel increase of the availability of deeper investigation which, as shown in this report, is as vital for molecular research as it is beneficial for clinical patient management.

## 6. Conclusions

In conclusion, we identified an exon 6–13 duplication in *CHEK2* in a patient tested for breast cancer susceptibility. We defined the extension, position and break-points of the rearrangement, and performed a quantitative RNA analysis to assess variant pathogenicity. These results highlight the importance of molecular characterizations and functional studies on intragenic rearrangements, especially in genes with high rates of VUS, such as *CHEK2*.

## Data Availability

All data will be available upon reasonable request at the corresponding author.
